# Label-free quantitative shotgun analysis of bis(monoacylglycero)phosphate lipids

**DOI:** 10.1007/s00216-025-05890-4

**Published:** 2025-05-09

**Authors:** Lian Y. Wang, Rico J. E. Derks, Kevin A. J. Brewster, Danilo Prtvar, Sabina Tahirovic, Stefan A. Berghoff, Martin Giera

**Affiliations:** 1 Center for Proteomics and Metabolomics, Leiden University Medical Center, Albinusdreef 2, Leiden, 2333ZA The Netherlands; 2 German Center for Neurodegenerative Diseases (DZNE), Munich, 81377 Germany; 3 Institute of Neuronal Cell Biology, Technical University Munich, Munich, Germany

**Keywords:** BMP, Mass spectrometry, Shotgun lipidomics, Label free, Flow injection

## Abstract

Interest in the role of bis(monoacylglycero)phosphate (BMP) lipids in lysosomal function has significantly grown in recent years. Emerging evidence highlights BMPs as critical players not only in Niemann-Pick disease type C (NPC) but also in other pathologies such as neurodegeneration, cardiovascular diseases, and cancers. However, the selective analysis of BMPs is significantly hindered by isomeric phosphatidylglycerol (PG) lipids. While this can be addressed by chromatographic separation, it poses a significant challenge for shotgun lipidomics approaches. Here, we present a shotgun lipidomics strategy to detect and separate BMPs from PGs using differential fragmentation of sodiated ions. This approach, including isotope correction, is integrated into an existing quantitative shotgun lipidomics workflow (Lipidyzer combined with Shotgun Lipidomics Assistant software) that simultaneously quantifies >1400 lipids. Validation using K-562 cell extracts demonstrated acceptable linearity, trueness, repeatability, and a limit of quantification of 0.12 µM, confirming robust analytical performance. Finally, characteristic accumulation of BMP lipids is shown in bone marrow–derived macrophages from NPC mice, demonstrating its applicability. Our method presents a quantitative, selective, rapid, and robust solution for shotgun-based BMP analysis without the need for extensive chromatographic separation or derivatization. The integration of BMP lipid detection into the Lipidyzer platform, alongside the recently launched iSODA data visualization tool, empowers chemists and biologists to gain deeper insights into BMP lipid biology.

**Graphical Abstract:**

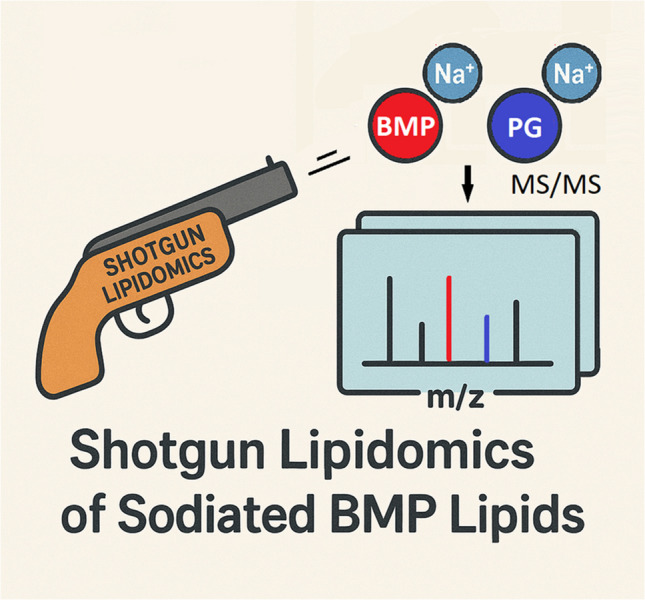

**Supplementary information:**

The online version contains supplementary material available at 10.1007/s00216-025-05890-4.

## Introduction

Lipidomics has emerged as a promising tool applied in various disciplines, such as cardiovascular disease, oncology, and neurodegeneration [1–4]. The detailed study of lipids typically requires a combination of advanced technologies owed to their large chemical and biological heterogeneity [1, 3–6]. One such technology, shotgun lipidomics, measures lipids on either high-resolution or triple quadrupole mass spectrometry instruments using direct infusion or flow injection approaches, where lipids are predominantly separated through characteristic mass spectrometric fragmentations [7]. An advanced quantitative shotgun lipidomics approach, employing differential ion mobility spectrometry (DMS) for enhanced lipid class separation, combined with multiple reaction monitoring (MRM) on a triple quadrupole (or QTrap) mass spectrometer, was initially developed by SCIEX (Lipidyzer™) and subsequently rendered open access through the publication of the Shotgun Lipidomics Assistant (SLA) software solution [1, 8]. This approach has widely become recognized as a quantitative and reproducible assay for the wide targeted analysis of lipids [8–10]. However, given its targeted character, the platform only quantifies predefined lipid classes and species. Recently, several lipid classes, as for example cardiolipins and phosphatidic acids [11, 12], have been added to the platform. Yet, other important classes, such as bis(monoacylglycero)phosphate (BMP) lipids, are not yet incorporated for quantitative shotgun lipidomics analysis. BMPs are expressed almost exclusively in lysosomal bodies, where they are believed to facilitate healthy (lipid) metabolism during homeostasis [2]. They have been linked to a multitude of diseases, including but not limited to lysosomal storage disorders, cancers, and neurodegeneration, indicating a crucial role of BMPs in lysosomal function [2–4, 13–15]. These results have sparked increasing demand for the rapid and quantitative analysis of these lipids.

Analytically speaking, BMPs pose a couple of specific challenges related to their quantitative analysis, with the most important one being their distinction from isomeric phosphatidylglycerol (PG) lipids. To overcome this issue, most analytical strategies rely on chromatographic separation of these isobaric lipid classes [3, 16]. Typical liquid chromatography mass spectrometry (LC–MS)–based platforms make use of reversed-phase chromatography and extended gradients to accomplish lipid separation, although alternative separation solutions have also been demonstrated to be possible [17, 18]. While these approaches allow for the selective analysis of BMPs, the prolonged run times required by extended LC gradients make them less suitable than shotgun lipidomics for high-throughput applications in large-scale lipidomic studies. Alternatively, a derivatization-based solution compatible with shotgun lipidomics analysis was developed by the group of Han, which developed a one-step methylation strategy, allowing separation of PGs and BMPs [5]. While this approach allows for the rapid and selective shotgun analysis of BMPs, the necessary derivatization step limits its applicability as part of a larger lipidomics panel.

In summary, none of the current approaches are practical for the integration of BMP into a large panel of lipids as analyzed by the Lipidyzer platform. However, integrated analysis of BMP is desirable for multiple reasons. Re-analysis and re-injection of samples are avoided, batch effects are reduced, and uniform comparable data across the lipidome is obtained, ultimately leading to more robust conclusions. To overcome these issues and enable the facile analysis of BMP lipids as part of a large panel of lipid classes, we here set out to develop a label-free quantitative shotgun lipidomics approach for the selective analysis of BMP lipids. The developed strategy was integrated into the Lipidyzer platform and the SLA workflow software, including isotope correction.

To guarantee selective, quantitative, and accurate analysis of BMP lipids, separation from isobaric PG lipids is paramount. Derivatization-independent strategies enabling this separation during shotgun lipidomics analysis potentially include lipid class separation using ion-mobility approaches such as DMS or the use of selective tandem mass spectrometric (MS/MS) transitions. As shown here, derivatization-independent, rapid, robust, quantitative, and selective shotgun lipidomics analysis of BMP lipids is possible when sodiated molecular ions in positive mode electrospray ionization (ESI +) are used for collision-induced dissociation (CID).

## Materials and methods

### Materials

Methyl tert-butyl ether (MTBE) was obtained from Sigma-Aldrich, methanol (MeOH), and ammonium acetate were from Supelco. Dichloromethane (DCM), LC–MS grade water, and 1-propanol were sourced from Honeywell, Riedel de Haën.

The following lipid IS and unlabeled BMP standards were purchased from Avanti (Alabaster, AL, USA): UltimateSPLASH One, dFA 18:1-d9, dCER d18:0 – d7/13:0, GluCER d18:1 – d7/15:0, LacCER d18:1 – d7/15:0, PA 15:0–18:1-d7, BMP 14:0/14:0, BMP 18:1/18:1. Additionally, “EquiSPLASH LIPIDOMIX Quantitative Mass Spec Internal Standard mix” was purchased from Avanti for use in DMS cell tuning.

### K-562 cell culture

K-562 cells were cultured in T75 NUNC EasyFlask flasks in Gibco IMDM (1 ×) medium (Gibco, 12,440–053) supplemented with 0.2% penicillin–streptomycin (Gibco, 15,140–122) and 10% Gibco fetal bovine serum, heat inactivated (Gibco, A52568-01). Cells were cultured until 80% confluency and harvested using trypsin (Wisent, 325–542-EL). Cell density was calculated by placing 10 µL of the cell suspension into a dual-chamber cell counting slide (Bio-Rad, 1,450,003, USA), which was then analyzed using the TC20 automated cell counter (Bio-Rad, 1450102, USA). The pellet was subsequently spun down at 300 × g for 5 min at room temperature (RT). After discarding the supernatant and a brief phosphate-buffered saline (PBS) washing step, samples were spun down again at 300 × g for 5 min at RT. After discarding the supernatant, the pellets were resuspended in PBS to reach a concentration of 40 million cells/mL. Finally, 25 µL aliquots were created (1 million cells/sample).

### NPC1 cKO mice-derived bone marrow–derived macrophages

BMDMs were isolated from the femora of adult NPC1 cKO mice generated as described elsewhere [19] by crossing the Npc1 flox/flox line with the CX3 CR1-Cre line [20, 21]. Cells (5 × 10^5^) were plated in 10-cm-diameter tissue culture dishes in L929-conditioned media (20%). For experimental seeding, cells were cultivated (DMEM, 1% penicillin/streptomycin and 10% fetal calf serum) at 37 °C with 7.5% CO_2_. For activation, cells were treated with 0.1% lipid mix (Sigma; L0288-100ML) for 16 h before harvesting for lipidomic analysis. The Npc1flox line (CXrCR1-) was used as a control.

### Internal standard preparation

BMP 14:0/14:0 was added to the current Lipidyzer internal standard mix at a concentration of 4 µg/mL (6.02 µM) [22].

### Lipid extraction

Extraction of lipids was achieved using MTBE, similar to the protocol described in Ghorasaini et al. and Matyash et al. [9, 10, 23]. In brief, after the addition of the BMP supplemented IS mix, samples are mixed with a mixture of MTBE and MeOH. Cells are subsequently lysed through brief (~ 2 min) ultra sonification at maximum intensity. After letting samples rest for approximately 30 min and centrifugation (5 min, 18,213 × g), the supernatant is collected into a fresh Eppendorf vial. After a second extraction, the supernatants from both extractions are combined and mixed with LC–MS water, forming a biphasic solution. After centrifugation (5 min, 18,213 × g), lipids are collected from the upper layer, which is subsequently dried using a gentle nitrogen stream before being reconstituted in “lipidomics running buffer” (50:50 DCM:MeOH + 10 mM ammonium acetate).

### MS/MS analysis

Samples were measured using a SCIEX QTRAP 6500+ mass spectrometer fitted with a SelexION DMS device. 1-Propanol was used as DMS modifier gas. Samples were administered through flow injection using a Shimadzu LC-30 autosampler with lipidomics running buffer as the liquid phase. Optimal separation settings for the DMS device were tuned on the day of analysis using equiSPLASH LIPIDOMIX. All samples were measured in MRM mode using both a shotgun lipidomics method and a DMS lipidomics method. BMP MRM values were integrated into the shotgun lipidomics method. A comprehensive list of MS settings can be found in the Supplementary Table S1 and reference [8]. BMP lipids were added to “method2” of the Lipidyzer platform operating without DMS separation. Details about the mass spectrometric fragmentation of BMP and PG lipid can be found in the results section.

### Lipidyzer analysis

Raw data outputs from Analyst are read by SLA [8]. Using BMP-supplemented dictionaries (Supplementary Table S2-S4), SLA applies various corrections to the data. Among these are checks for data quality and correction for naturally occurring isotopes. In addition, SLA calculates the median intensities from the measured MS cycles (*n* = 20) and compares them to the median intensities of specified IS, resulting in analyte concentrations. Resulting data was further analyzed using SODA light, a lightweight branch of the integrated data analysis tool iSODA [24] used by the Neurolipid Atlas [22] (www.neurolipidatlas.com). iSODA filters for features that exceed blank signals by at least twofold and applies normalization to the data based on total measured lipid content per sample in % of total lipidome.

### Validation experiments

Validation experiments were conducted by spiking 25 µL aliquots of K-562 cells, each containing 1 million cells. BMP was added to samples using BMP dissolved in a 50:50 MeOH/DCM solution. For the selectivity experiments, 10 µL of 100 µg/mL BMP 18:1/18:1 (51.7 µM), 10 µL of 100 µg/mL PG 18:1/18:1 (51.7 µM), or 50 µL of 100 µg/mL PG 18:1/18:1 (258.7 µM) was added to K-562 aliquots.

Calibration lines for K-562 cell extracts (25 µL, 1 million cells) and LC–MS water (25 µL) were generated in parallel. BMP 18:1/18:1 of differing concentrations, dissolved in a 50:50 MeOH/DCM solution, was added to K-562 cell aliquots or LC–MS water as follows: 10 µL of 5 µg/mL, 10 µL of 10 µg/mL, 50 µL of 10 µg/mL, 10 µL of 100 µg/mL, 20 µL of 100 µg/mL, and 50 µL of 100 µg/mL (*n* = 3). Linearity and slopes were calculated using linear regression. The repeatability of BMP 18:1/18:1 spiking was assessed across five independent experiments through the addition of 10 µL of 10 µg/mL or 10 µL of 100 µg/mL BMP 18:1/18:1 dissolved in a 50:50 MeOH/DCM solution (*n* = 5).

## Results and discussion

### DMS-based lipid class separation and selectivity assessment

**Fig. 1:**
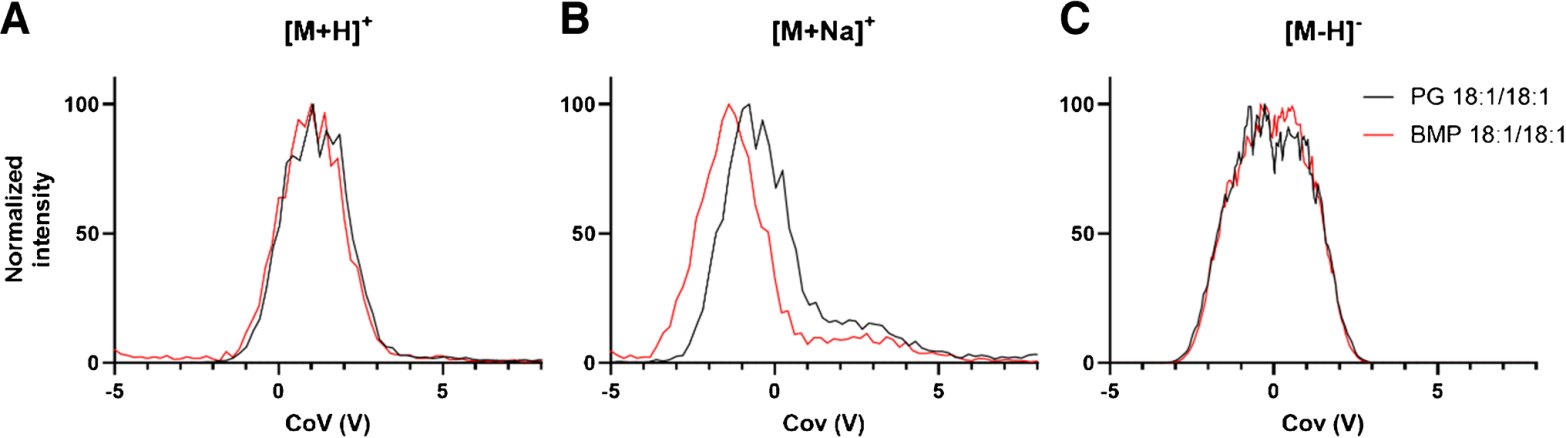
Differential mobility behavior across ramped COV compensation voltages (V) of several PG 18:1/18:1 and BMP 18:1/18:1 ions at a separation voltage of 3800 V

As a starting point, we tested the DMS-based separation of both PG and BMP lipid classes using a pair of isobaric BMP and PG species (BMP 18:1/18:1 and PG 18:1/18:1) in ESI + and ESI- modes, investigating their [M + H]^+^, [M + NH_4_]^+^, [M + Na]^+^, and [M - H]^−^ ions. However, as shown in Fig. 1 and also partially described by Hankin et al. [6], DMS separation is insufficient for the separation of the tested isobaric PG and BMP lipids. Moreover, [M + NH4]^+^ ions became undetectable after the DMS cell was turned on and were thus excluded from subsequent analyses.

**Fig. 2:**
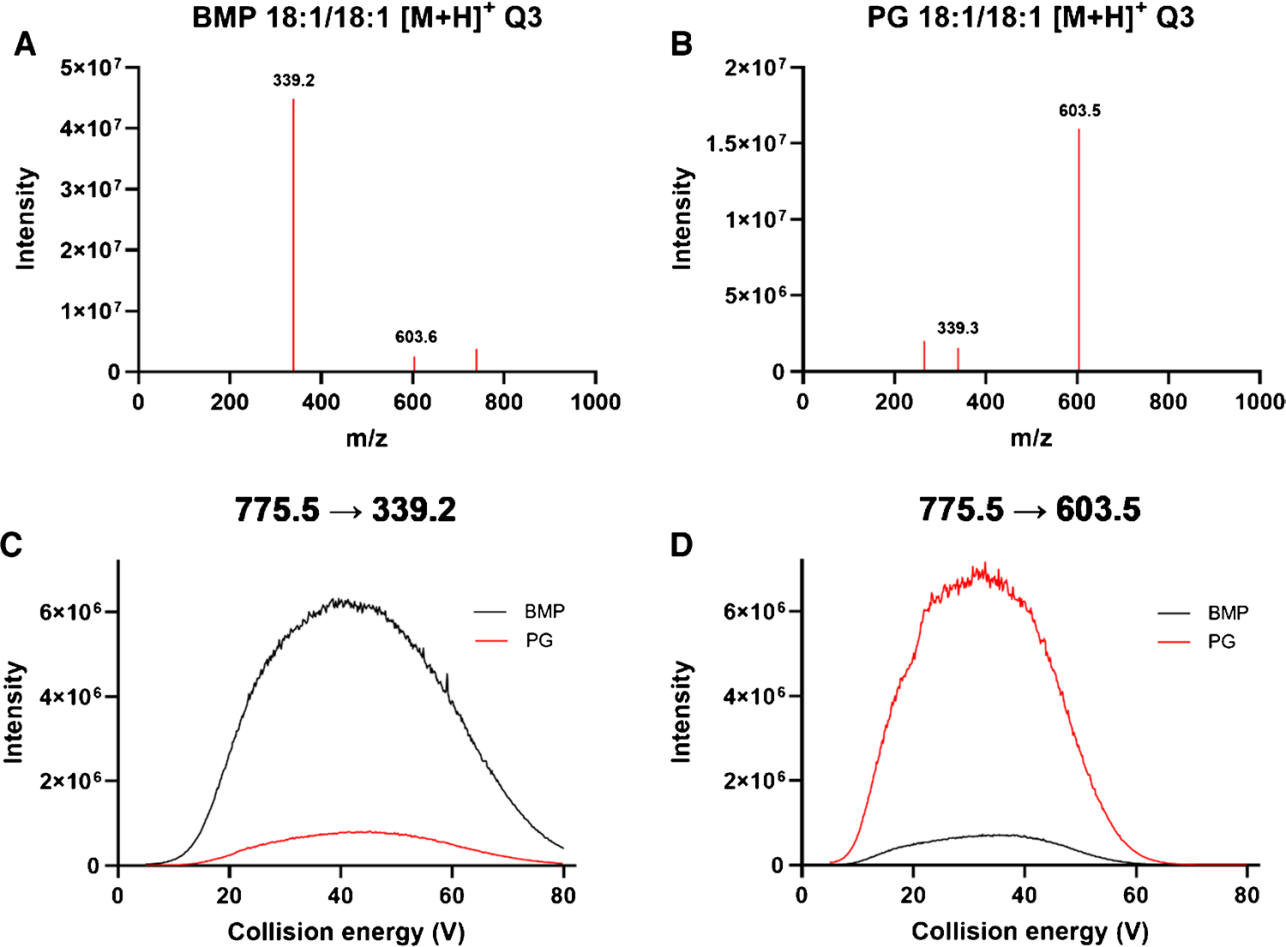
Fragmentation spectra of the precursor ion *m/z* 797.5 for **A** BMP 18:1/18:1 and **B** PG 18:1/18:1. **C** Monitoring the transition *m/z* 775.5 → 339.2 while infusing BMP (16.7 µg/mL) (black line) and PG (16.7 µg/mL) (orange line) and ramping the collision energy. **D** Monitoring the transition *m/z* 775.5—> 603.5 while infusing BMP (16.7 µg/mL) (black line) and PG (16.7 µg/mL) (orange line) and ramping the collision energy

As DMS-based separation of PG and BMP was insufficient, we next aimed at leveraging selective MS/MS fragments. To this end, the group of Murphy [6] has described MS/MS fragmentation of both lipid classes in detail, pointing out that CID of [M - H]^−^ ions leads to identical MS/MS spectra. On the other hand, CID of [M + H]^+^ and [M + NH_4_]^+^ ions leads to fragment ions enriched in either one of the lipid classes. For BMP 18:1/18:1 and PG 18:1/18:1, this specifically means that BMP lipids primarily dissociate into a monoacyl-group fragment corresponding to a dissociation at the phosphate group, resulting in a fragment ion *m/z* 339 for BMP 18:1/18:1 (Fig. 2A, supplementary Figure S1), whereas PG lipids primarily dissociate into a diacyl-group fragment, resulting in a fragment ion of *m/z* 603 for PG 18:1/18:1 (Fig. 2B, supplementary figure S2).

Nevertheless, while the fragmentation spectra of both lipid classes are enriched in these fragments, there is still considerable overlap between the two. In turn, the employment of these ions/transitions during shotgun lipidomics analysis would consequently lead to contamination between the two lipid classes. We evaluated the overlap between the *m/z* 775.5 → 339.2 and *m/z* 775.5 → 603.5 mass transitions in ESI + MRM mode through the infusion of BMP 18:1/18:1 (16.7 µg/mL) and PG 18:1/18:1 (16.7 µg/mL) while simultaneously assessing the influence of collision energy on the formation of the *m/z* 339.2 or 603.5 fragments (Fig. 2C–D). At the collision energy with optimal sensitivity (Fig. 2C, collision energy = 39.6 V), PG lipids would account for roughly 12% of the BMP trace at equimolar concentration when using the *m/z* 775.5 → 339.2 transition. Furthermore, the results did not reveal a specific collision energy that would allow for selective analysis of the lipid classes. Although the contamination may appear minor, it can potentially lead to biological misinterpretations, particularly in extreme situations, such as bacterial extracts, which can contain disproportionate amounts of PG compared to BMP lipids [25].

Next, as DMS was insufficient for the separation of isobaric BMP and PG lipids, we searched for alternative solutions. While investigating, we observed a significant fraction of sodiated ions in the ESI + spectra of BMP (and PG) lipids (supplementary Figure S3-S5). Importantly, unlike fragmentation of [M + H]^+^ and [M + NH_4_]^+^ ions, [M + Na]^+^ ions give rise to lipid class-selective fragments that we subsequently investigated for their applicability in label-free, selective, and quantitative shotgun lipidomics analysis of BMP lipids. The consistent formation of [M + Na]^+^ BMP ions was tested in both LC–MS grade water and K-562 cell extract for two BMP lipid species, BMP 18:1/18:1 (16.7 µg/mL) and BMP 14:0/14:0 (16.7 µg/mL) (supplementary Figure S6). Overall, the general distribution of molecular adduct ions remained stable when comparing the two matrices and lipid species. In summary, DMS as well as CID fragmentation of [M - H]^−^ and [M + H]^+^ is insufficient for the selective analysis of BMP using shotgun lipidomics analysis. However, CID-based fragmentation of [M + Na]^+^ ions gives rise to selective fragments that can be used for quantitative shotgun lipidomics analysis of BMP lipids. The specific fragmentation routes for sodiated BMP 18:1/18:1 and PG 18:1/18:1 are shown in supplementary Figure S7 and can also be found in Hankin et al. [6]. The fragmentation spectra for sodiated BMP 18:1/18:1 and PG 18:1/18:1 are shown in Figure S8.

### Shotgun analysis using sodiated ions

**Fig. 3:**
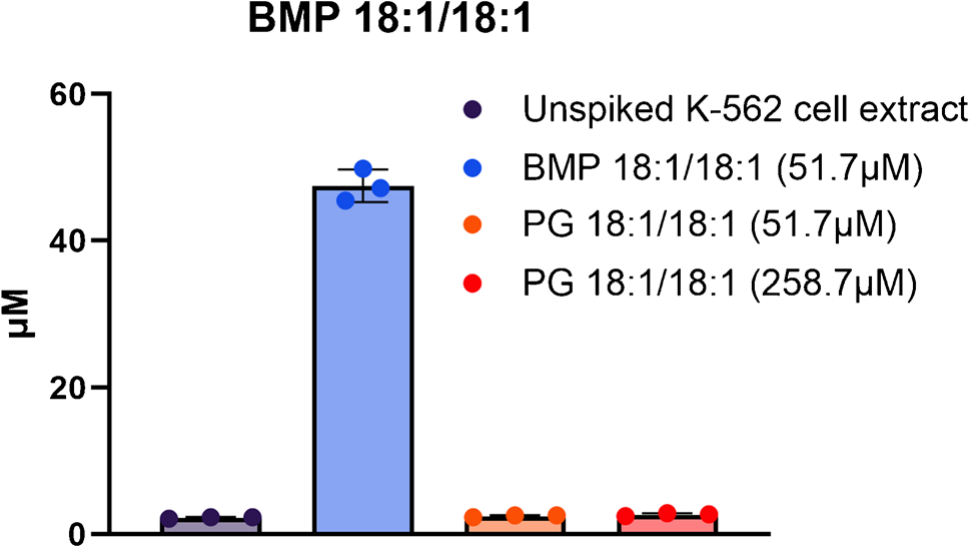
Selectivity assessment for the analysis of sodiated BMP 18:1/18:1

As a first and most important step in this investigation, we established selectivity for BMP analysis, isolating and fragmenting sodiated molecular ions. As we had established that DMS is not sufficiently separating isobaric BMP and PG lipids, we carried out all experiments without applying DMS. We spiked K-562 cell extract samples with either 40 µg/mL BMP 18:1/18:1 (51.7 µM) or a low/high concentration of 40/200 µg/mL (51.7 µM/248.7 µM) PG 18:1/18:1 and quantified the BMP lipid against an external calibration line. After spiking, BMP 18:1/18:1 concentrations increased correspondingly, whereas the observed BMP levels were completely unaffected by PG spiking at two different concentration levels (Fig. 3), indicating that there was no significant cross talk between the traces when using sodiated molecular ions and lipid class selective fragments.

Next, we assessed the quantitative character of the method using BMP 18:1/18:1 as a surrogate BMP lipid. During the development of the method, no deuterated BMP standards were commercially available. In turn, and as also done by others, we used BMP 14:0/14:0 as the internal standard (IS). To obtain accurate quantitative results during shotgun lipidomics analysis, isotope correction is mandatory. Isotope correction was integrated using the SLA built-in isotope correction library, which was expanded with BMP-specific parameters, calculated using Rdisop, identical to the SLA guidelines (Supplementary Table S2) [8]. The dictionaries for integrating the BMP lipid class into the existing Lipidyzer platform [9, 10] can be found in Supplementary Table S2–S4. To assess linearity, as well as process efficacy [26] we spiked increasing amounts of BMP 18:1/18:1 into K562 cell extracts or water blanks and compared the slopes of the calibration lines to theoretical trueness. The calibration line was also used to calculate the lower limit of quantification (LLOQ), defined as the mean blank concentration + 10 * SD of the blanks. Similarly, the limit of detection (LOD) was calculated as the mean blank concentration + 3.3 * SD of the blanks, where LC–MS grade water served as the blank. Finally, we assessed intra- and inter-day repeatability in K-562 cell extracts across five independent batches, as well as trueness. A summary of our validation results is shown in Table 1.

**Table 1:** Analytical figures of merit

Analytical parameter	Result
Calibration range	0–258.7 µM
Calibration function	
Synthetic standard solution	*y = 42.40x – 1.795*
Spiked cell extract	*y* = 34.14*x* – 1.216
Linearity (*R*^2^)	
Synthetic standard solution	0.9932
Spiked cell extract	0.9925
Lower limit of quantification (LLOQ)	0.12 µM
Limit of detection (LOD)	0.04 µM
Intra-day repeatability (*n* = 5), RSD	
0.1 µg/mL	5.6%
1 µg/mL	4.9%
Inter-day repeatability (*n* = 5), RSD	
0.1 µg/mL	10.7%
1 µg/mL	14.8%
Trueness (measured/expected) intra-day (*n* = 5)	
0.1 µg/mL	96.3% ± 2.53%
1 µg/mL	98.1% ± 3.81%
Trueness (measured/expected) inter-day (*n* = 5)	
0.1 µg/mL	85.9% ± 9.23%
1 µg/mL	83.5% ± 12.33%

*RSD* refers to relative standard deviation. *LLOQ* was calculated as “mean blank concentration + 10 * SD of the blanks.” *LOD* was calculated as “mean blank concentration + 3.3 * SD of the blanks.” Blank refers to LC–MS grade water, which was worked up according to the Lipidyzer protocol (see the “Materials and methods” section)

### Application in biological studies

**Fig. 4:**
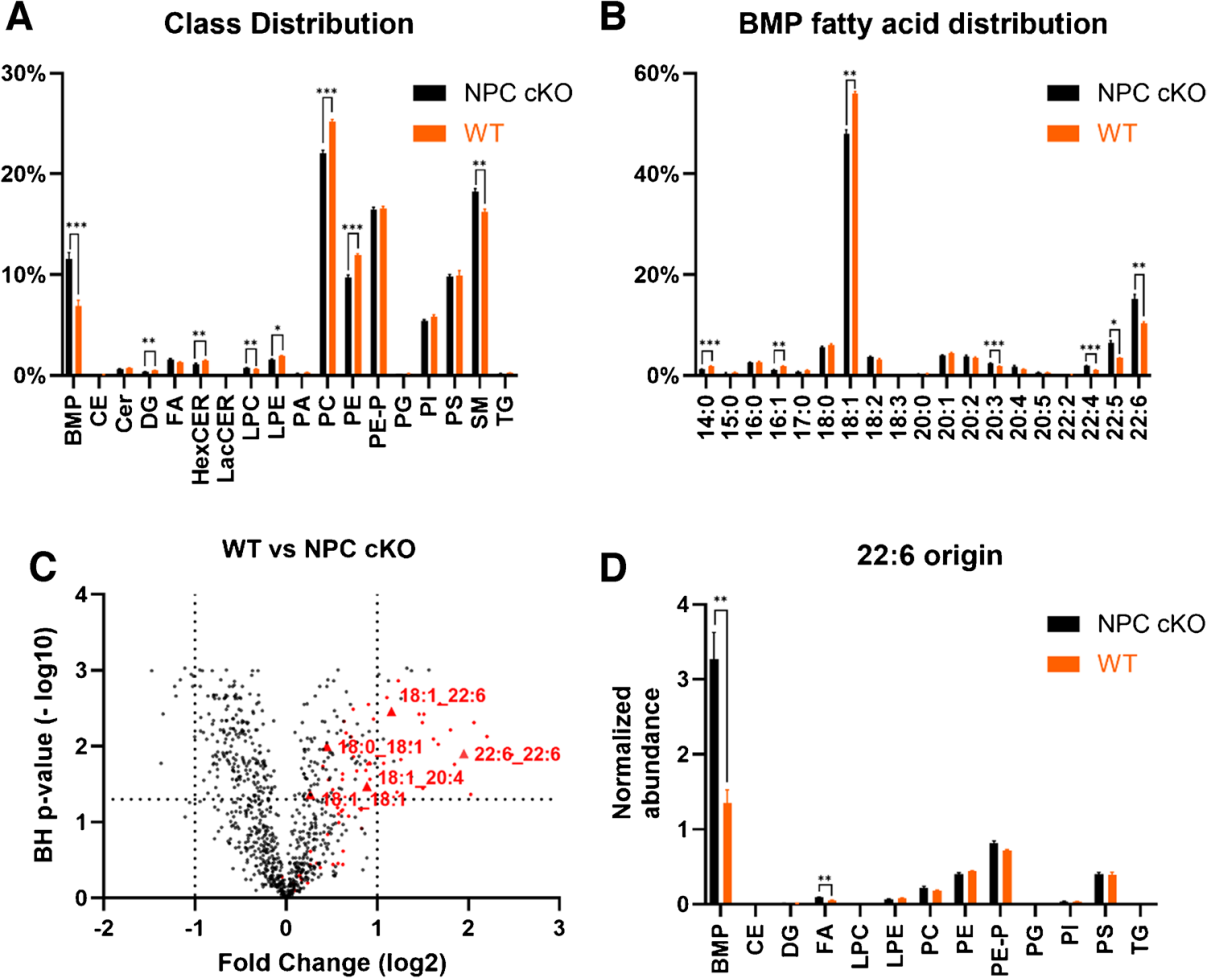
Integrated wide targeted lipidomic analysis of NPC1 cKO bone marrow-derived macrophages. **A** Relative distribution of measured lipid classes. Lipid concentrations were normalized against the total sum of lipids in each sample (*n* = 4). **B** Relative distribution of FA tails in BMP lipids. FA concentrations are normalized against the total sum of BMP FA in each sample (*n* = 4). **C** Volcano plot of individual lipid species. BMP lipids are colored in red. Notable abundant BMP lipids are annotated. *p*-values are calculated using an unpaired Welch *t* test with BH correction (*n* = 4). **D** FA 22:6 distribution across all monitored lipid classes. Lipid concentrations, normalized to total lipid contents, were converted into normalized FA abundance. *p < 0.05; **p < 0.01; ***p < 0.001

Finally, we assessed the biological applicability of the method as part of a large lipid panel in an in vivo model. Accumulation of BMP lipids is a critical hallmark of Niemann Pick disease type C (NPC) and can be used as a diagnostic biomarker [2, 14, 15, 27]. In turn, we modeled NPC using a recently generated mouse model with a depletion of NPC1 in myeloid cells [19] and compared bone marrow–derived macrophages (BMDM) from NPC1 conditional knockout mice (NPC1 cKO) with control BMDMs. The results are shown in Fig. 4 or can alternatively be viewed dynamically in the NeuroLipid Atlas (NLA) (www.neurolipidatlas.com, see the “Data Availability” section for details) [22]. NLA pretreats data before analysis [22]. Briefly, it filters relevant lipid species through comparison with blank samples and removes all lipid species not reaching a level of > 2 times the concentration in blanks. Subsequently, the data browser calculates the concentration of fatty acids (FA) from each lipid species, accounting for the difference in number of FA per lipid class. As the exact composition of a triglyceride (TG), which contains three FA’s, cannot be determined using the current setup, the composition of TGs is expressed as total carbon and double bond number, specifying the identified FA, e.g., TG 60:4–18:1.

BMP lipid levels are strongly dependent on species and/or tissue type [2]. Here, we show a relative contribution of 6.9% of BMP to the total lipidome of murine BMDMs, which is increased to 11.5% (1.6-fold increase, unpaired Welch *t* test, Benjamini-Hochberg (BH) corrected: *p* = 0.0008) in NPC1 cKO (Fig. 4A). The distribution of FA tails in the BMP class is similar to previous reports, containing high proportions of 18:1 and 22:6 FAs (Fig. 4B) [2]. Although the BMP class as a whole is upregulated in NPC1 cKO, individual species appear to be differentially affected (Fig. 4B–C). Specifically, the accumulation of BMP lipids containing FA 22:6 tails is particularly pronounced in NPC1 cKO BMDM (2.42-fold increase, unpaired Welch *t* test, BH corrected *p* = 0.0096), closely resembling earlier clinical reports [28]. Notably, FA 22:6 concentrations are unaffected in other lipid classes (Fig. 4D), indicating an increase in total FA 22:6 concentrations and not a redistribution. This seems remarkable as endogenous concentrations of highly unsaturated FAs are believed to predominantly stem from dietary uptake rather than endogenous biosynthesis.

## Discussion

Here, we describe the label-free, rapid, accurate, and quantitative shotgun analysis of BMP lipids, integrated with an existing large panel of lipid classes monitored by flow-injection DMS-MS/MS analysis (Lipidyzer, 17 lipid classes, >1400 lipid species). Analysis of the entire panel of more than 1400 lipid species takes approximately 30 min; however, selected analysis of the BMP class can be done in as little as 75 s per sample, providing significant throughput. BMP lipids can accurately be quantified by fragmenting sodiated ions, thereby generating specific fragments for both BMP and isomeric PG lipids. As an IS, we had to reside with BMP 14:0/14:0, as no isotopically labeled IS was commercially available at the time of development. However, as BMP 14:0/14:0 is generally not present in biological materials, this did not affect method performance, as can be argued by obtaining highly accurate results when spiking cell extracts with BMP 18:1/18:1. Nevertheless, particularly for the accurate quantification of highly unsaturated BMP, the use of a more closely related isotopically labeled BMP IS would be preferred in the future. The quantitative nature of the presented assay can be underlined by acceptable analytical figures of merit (Table 1). The obtained sensitivity (LLOQ = 0.12 µM) was sufficient for detecting changes in BMP metabolism in a murine NPC1 model and proves comparable to other published methods [29]. Linearity was assessed from 0 to 258.7 µM, and acceptable results were obtained (Table 1). Trueness was found to be somewhat affected by the matrix, as judged from a slight reduction of the calibration slope when comparing neat with matrix-fortified extracts. Nevertheless, as the presented method is not aimed at critical clinical decision making but rather as a high-throughput research tool, this seemed acceptable to us. Furthermore, some variance is presumably inherent to the Lipidyzer platform itself, rather than the measurements of BMP lipids. To obtain accurate quantitative results, we incorporated isotope correction using the Rdisop package.

The addition of BMP lipids significantly broadens the applicability of the Lipidyzer workflow and can be used by researchers in various scientific fields to reveal distinct properties of BMP lipids in direct comparison to other lipid classes. For example, BMP lipids express a very distinct FA composition. The overenrichment of FA 22:6, presumably n3-docosahexaenoic acid (DHA), in BMP lipids is most notable. Despite BMP lipid concentrations composing only 6.9% (Fig. 4A) of total measured lipid concentration in WT samples, they harbor 41.2% (Fig. 4D) of the total measured pool of FA 22:6, which in turn composes only 1.71% of all measured FAs (supplementary Figure S9). Furthermore, this is in direct contrast to their proposed precursor PG, which is practically devoid of FA 22:6 (Fig. 4D). The overenrichment of FA 22:6 can also seemingly be differentially expressed, as demonstrated in the NPC1 cKO BMDM. Although the mechanism for this overenrichment remains unknown to date, this data suggests a specific role of FA 22:6 in BMP function. We are confident that the presented quantitative label-free shotgun lipidomics analysis of BMP lipids is a significant addition to the Lipidyzer toolbox and will enable researchers in various fields to gain deeper insights into this intriguing class of lipids.

## Conclusion

We here present a shotgun lipidomics strategy for the selective analysis of BMP lipids based on CID fragmentation of sodiated ions. Avoiding chromatographic separation as well as derivatization allowed for the integration of the BMP lipid class into the quantitative Lipidyzer platform. Together with the recently introduced iSODA [24] visualization tool, we are confident that this addition will allow for the robust quantitative analysis of BMP lipids and empower biologists and chemists to gain deeper insights into BMP lipid biology.

Supplementary information.

## Supplementary information

Below is the link to the electronic supplementary material.

ESM 1 (DOCX 496 KB)

ESM 2 (XLSX 8.93 KB)

ESM 3 (XLSX 228 KB

ESM 4 (XLSX 104 KB)

ESM 5 (XLSX 143 KB)

## Data Availability

A direct url to the NPC1 cKO dataset can be found here: https://cpm-lumc.shinyapps.io/soda-light/?experimentId=NLA_073.

## References

[1] Ubhi BK. Direct infusion-tandem mass spectrometry (DI-MS/MS) analysis of complex lipids in human plasma and serum using the Lipidyzer™ platform. Methods Mol Biol (Clifton, NJ). 2018;1730:227–36. 10.1007/978-1-4939-7592-1_15 29363076

[2] Medoh UN, Abu-Remaileh M. The bis(monoacylglycero)-phosphate hypothesis: from lysosomal function to therapeutic avenues. Annu Rev Biochem. 2024;93(1):447–69. 38603559 10.1146/annurev-biochem-092823-113814

[3] Logan T, Simon MJ, Rana A, Cherf GM, Srivastava A, Davis SS, et al. Rescue of a lysosomal storage disorder caused by Grn loss of function with a brain penetrant progranulin biologic. Cell. 2021;184(18):4651-68.e25. 34450028 10.1016/j.cell.2021.08.002 PMC8489356

[4] Akgoc Z, Sena-Esteves M, Martin DR, Han X, d’Azzo A, Seyfried TN. Bis(monoacylglycero)phosphate: a secondary storage lipid in the gangliosidoses. J Lipid Res. 2015;56(5):1006–13. 25795792 10.1194/jlr.M057851 PMC4409277

[5] Wang M, Palavicini JP, Cseresznye A, Han X. Strategy for quantitative analysis of isomeric bis(monoacylglycero)phosphate and phosphatidylglycerol species by shotgun lipidomics after one-step methylation. Anal Chem. 2017;89(16):8490–5. 28708380 10.1021/acs.analchem.7b02058

[6] Hankin JA, Murphy RC, Barkley RM, Gijón MA. Ion mobility and tandem mass spectrometry of phosphatidylglycerol and bis(monoacylglycerol)phosphate (BMP). Int J Mass Spectrom. 2015;378:255–63. 25883529 10.1016/j.ijms.2014.08.026 PMC4394388

[7] Han X, Gross RW. The foundations and development of lipidomics. J Lipid Res. 2022;63(2):100164. 10.1016/j.jlr.2021.100164 PMC8953652 34953866

[8] Su B, Bettcher LF, Hsieh WY, Hornburg D, Pearson MJ, Blomberg N, et al. A DMS shotgun lipidomics workflow application to facilitate high-throughput, comprehensive lipidomics. J Am Soc Mass Spectrom. 2021;32(11):2655–63. 34637296 10.1021/jasms.1c00203 PMC8985811

[9] Ghorasaini M, Mohammed Y, Adamski J, Bettcher L, Bowden JA, Cabruja M, et al. Cross-laboratory standardization of preclinical lipidomics using differential mobility spectrometry and multiple reaction monitoring. Anal Chem. 2021;93(49):16369–78. 10.1021/acs.analchem.1c02826 PMC8674878 34859676

[10] Ghorasaini M, Tsezou KI, Verhoeven A, Mohammed Y, Vlachoyiannopoulos P, Mikros E, et al. Congruence and complementarity of differential mobility spectrometry and NMR spectroscopy for plasma lipidomics. Metabolites. 2022;12(11):1030. 10.3390/metabo12111030 PMC9699282 36355113

[11] Riols F, Witting M, Haid M. Differential mobility spectrometry-based cardiolipin analysis. Methods Mol Biol (Clifton, NJ). 2025;2855:373–85. 10.1007/978-1-0716-4116-3_22 39354319

[12] Su B, Williams KJ. Analysis of the mammalian lipidome by DMS shotgun lipidomics. Methods Mol Biol (Clifton, NJ). 2025;2855:357–72. 10.1007/978-1-0716-4116-3_21 39354318

[13] Meikle PJ, Duplock S, Blacklock D, Whitfield PD, Macintosh G, Hopwood JJ, et al. Effect of lysosomal storage on bis(monoacylglycero)phosphate. Biochem J. 2008;411(1):71–8. 18052935 10.1042/BJ20071043

[14] Vanier MT, Gissen P, Bauer P, Coll MJ, Burlina A, Hendriksz CJ, et al. Diagnostic tests for Niemann-Pick disease type C (NP-C): a critical review. Mol Genet Metab. 2016;118(4):244–54. 27339554 10.1016/j.ymgme.2016.06.004

[15] Schuchman EH, Desnick RJ. Types A and B Niemann-Pick disease. Mol Genet Metab. 2017;120(1–2):27–33. 28164782 10.1016/j.ymgme.2016.12.008 PMC5347465

[16] Dominguez SL, Laufer BI, Ghosh AS, Li Q, Ruggeri G, Emani MR, et al. TMEM106B reduction does not rescue GRN deficiency in iPSC-derived human microglia and mouse models. iScience. 2023;26(11):108362. 37965143 10.1016/j.isci.2023.108362 PMC10641752

[17] Anderson DMG, Ablonczy Z, Koutalos Y, Hanneken AM, Spraggins JM, Calcutt MW, et al. Bis(monoacylglycero)phosphate lipids in the retinal pigment epithelium implicate lysosomal/endosomal dysfunction in a model of Stargardt disease and human retinas. Sci Rep. 2017;7(1):17352. 29229934 10.1038/s41598-017-17402-1 PMC5725462

[18] Scherer M, Schmitz G, Liebisch G. Simultaneous quantification of cardiolipin, bis(monoacylglycero)phosphate and their precursors by hydrophilic interaction LC−MS/MS including correction of isotopic overlap. Anal Chem. 2010;82(21):8794–9. 20945919 10.1021/ac1021826

[19] Colombo A, Dinkel L, Müller SA, Sebastian Monasor L, Schifferer M, Cantuti-Castelvetri L, et al. Loss of NPC1 enhances phagocytic uptake and impairs lipid trafficking in microglia. Nat Commun. 2021;12(1):1158. 33627648 10.1038/s41467-021-21428-5 PMC7904859

[20] Elrick MJ, Pacheco CD, Yu T, Dadgar N, Shakkottai VG, Ware C, et al. Conditional Niemann-Pick C mice demonstrate cell autonomous Purkinje cell neurodegeneration. Hum Mol Genet. 2010;19(5):837–47. 20007718 10.1093/hmg/ddp552 PMC2816612

[21] Yona S, Kim K-W, Wolf Y, Mildner A, Varol D, Breker M, et al. Fate mapping reveals origins and dynamics of monocytes and tissue macrophages under homeostasis. Immunity. 2013;38(1):79–91. 23273845 10.1016/j.immuni.2012.12.001 PMC3908543

[22] Feringa FM, Hertog SJK, Wang L, Derks RJE, Kruijff I, Erlebach L, et al. The Neurolipid Atlas: a lipidomics resource for neurodegenerative diseases uncovers cholesterol as a regulator of astrocyte reactivity impaired by ApoE4. bioRxiv. 2024. 10.1101/2024.07.01.601474.

[23] Matyash V, Liebisch G, Kurzchalia TV, Shevchenko A, Schwudke D. Lipid extraction by methyl-tert-butyl ether for high-throughput lipidomics. J Lipid Res. 2008;49(5):1137–46. 18281723 10.1194/jlr.D700041-JLR200 PMC2311442

[24] Olivier-Jimenez D, Derks RJE, Harari O, Cruchaga C, Ali M, Ori A, et al. iSODA: a comprehensive tool for integrative omics data analysis in single- and multi-omics experiments. Anal Chem. 2025;97(5):2689–97. 39886798 10.1021/acs.analchem.4c04355 PMC11822744

[25] Leeten K, Jacques N, Esquembre LA, Schneider DC, Straetener J, Henriksen C, et al. Ticagrelor alters the membrane of Staphylococcus aureus and enhances the activity of vancomycin and daptomycin without eliciting cross-resistance. mBio. 2024;15(10):e0132224. 39311589 10.1128/mbio.01322-24 PMC11481878

[26] Mazanhanga M, Joubert A, Castel S, Van de Merwe M, Maartens G, Wasserman S, et al. Validation of a quantitative liquid chromatography tandem mass spectrometry assay for linezolid in cerebrospinal fluid and its application to patients with HIV-associated TB-meningitis. Heliyon. 2023;9(11): e21962. 38034739 10.1016/j.heliyon.2023.e21962 PMC10685187

[27] Dinkel L, Hummel S, Zenatti V, Malara M, Tillmann Y, Colombo A, et al. Myeloid cell-specific loss of NPC1 in mice recapitulates microgliosis and neurodegeneration in patients with Niemann-Pick type C disease. Sci Transl Med. 2024;16(776):eadl4616. 39630885 10.1126/scitranslmed.adl4616

[28] Liu N, Tengstrand EA, Chourb L, Hsieh FY. Di-22:6-bis(monoacylglycerol)phosphate: a clinical biomarker of drug-induced phospholipidosis for drug development and safety assessment. Toxicol Appl Pharmacol. 2014;279(3):467–76. 24967688 10.1016/j.taap.2014.06.014

[29] Wang X, Schmitt MV, Xu L, Jiao Y, Guo L, Lienau P, et al. Quantitative molecular tissue atlas of Bis(monoacylglycero)phosphate and phosphatidylglycerol membrane lipids in rodent organs generated by methylation assisted high resolution mass spectrometry. Anal Chim Acta. 2019;1084:60–70. 31519235 10.1016/j.aca.2019.07.060

