# Supplementary material for: Label-free quantitative shotgun analysis of bis(monoacylglycero)phosphate lipids
Source: Anal Bioanal Chem. 2025 May 9;417(16):3665–73. doi: 10.1007/s00216-025-05890-4 (PMC12206189; doi:10.1007/s00216-025-05890-4)
Supplement: Supplementary file 1 — (DOCX 496 KB) [file 216_2025_5890_MOESM1_ESM.docx]

# Supplementary Materials


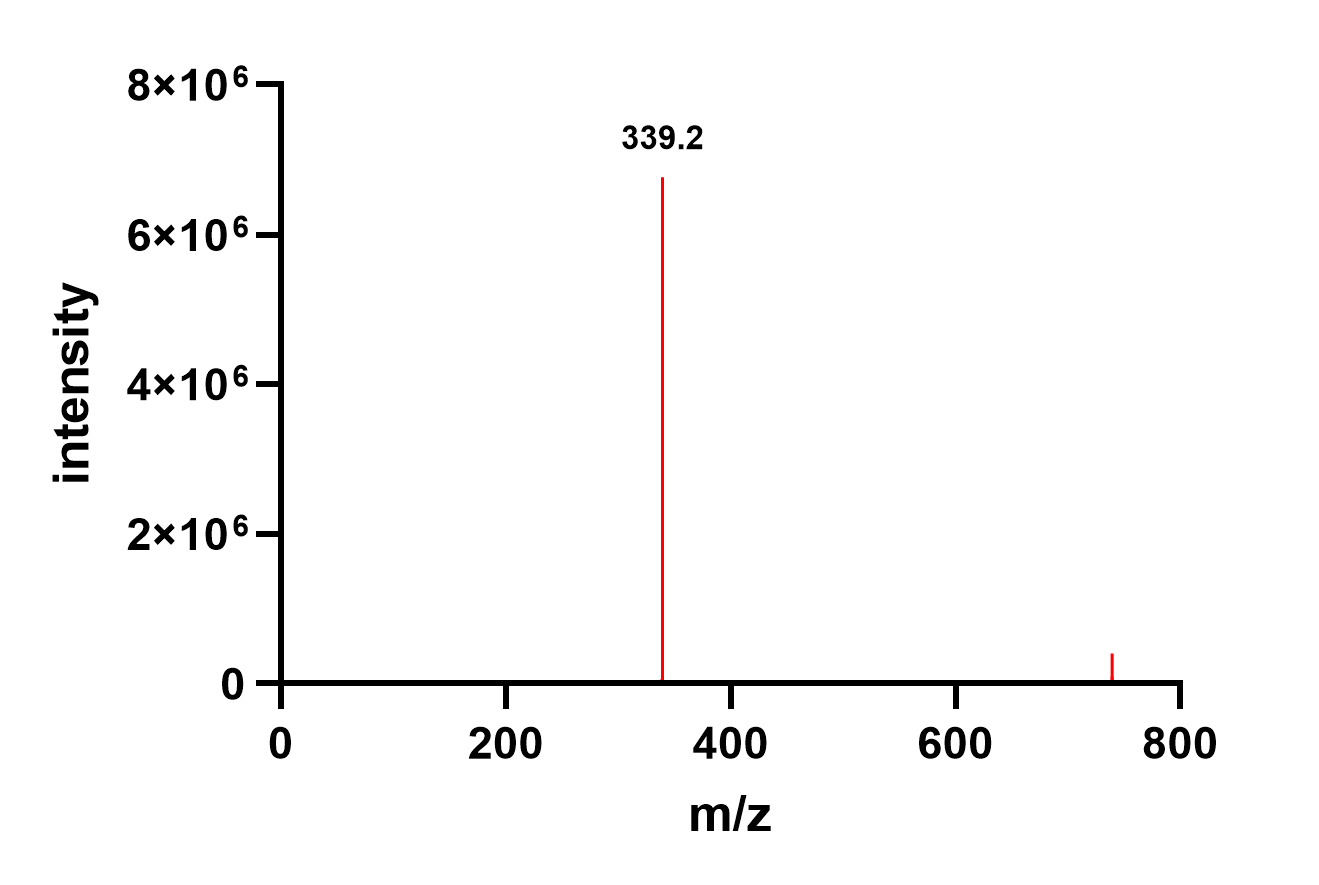


Figure S1: Fragments formed from BMP 18:1/18:1 + NH4^+^ ions (m/z = 792.5)


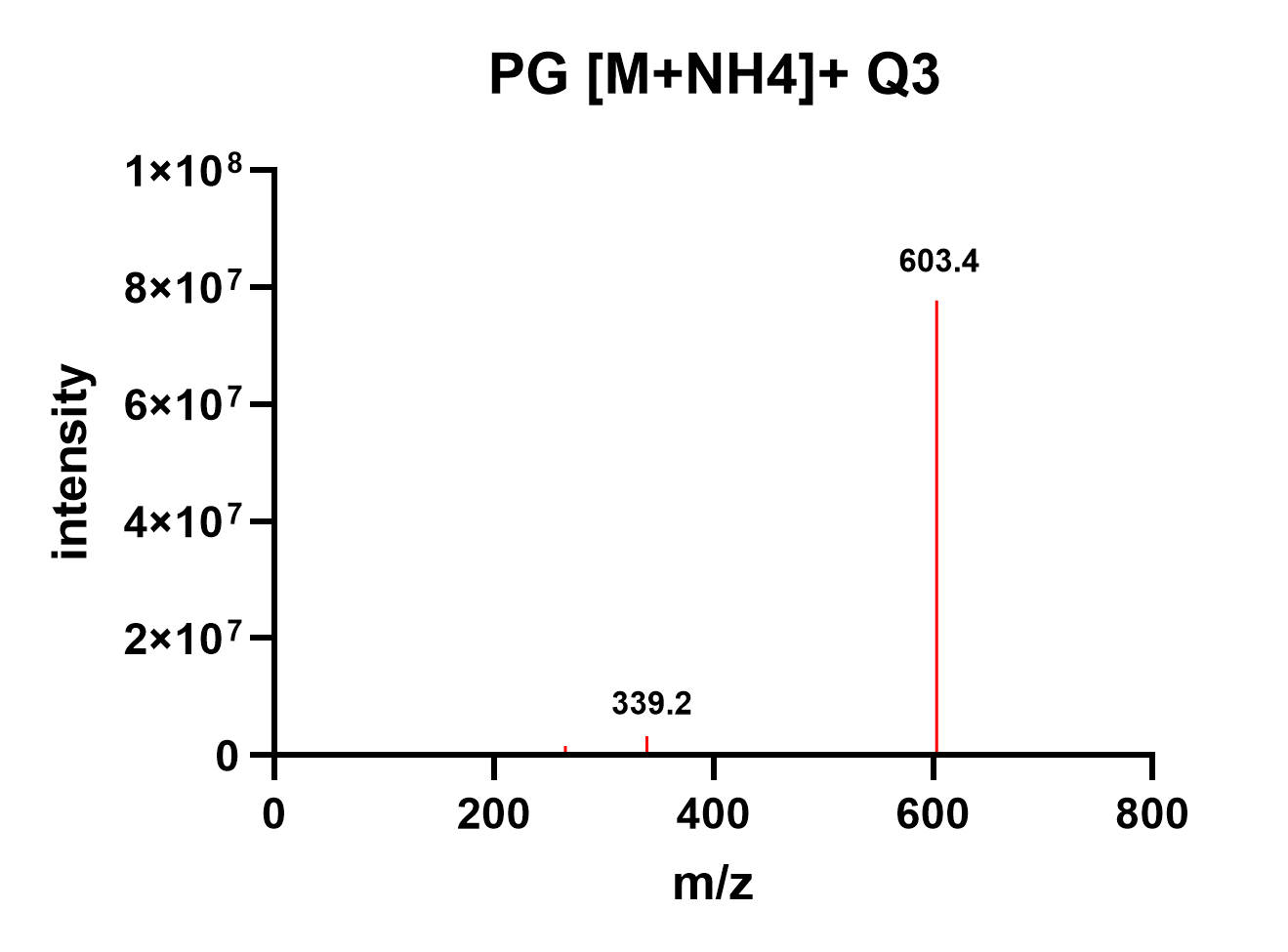


Figure S2: Fragments formed from PG 18:1/18:1 + NH4^+^ ions (*m/z* = 792.5)


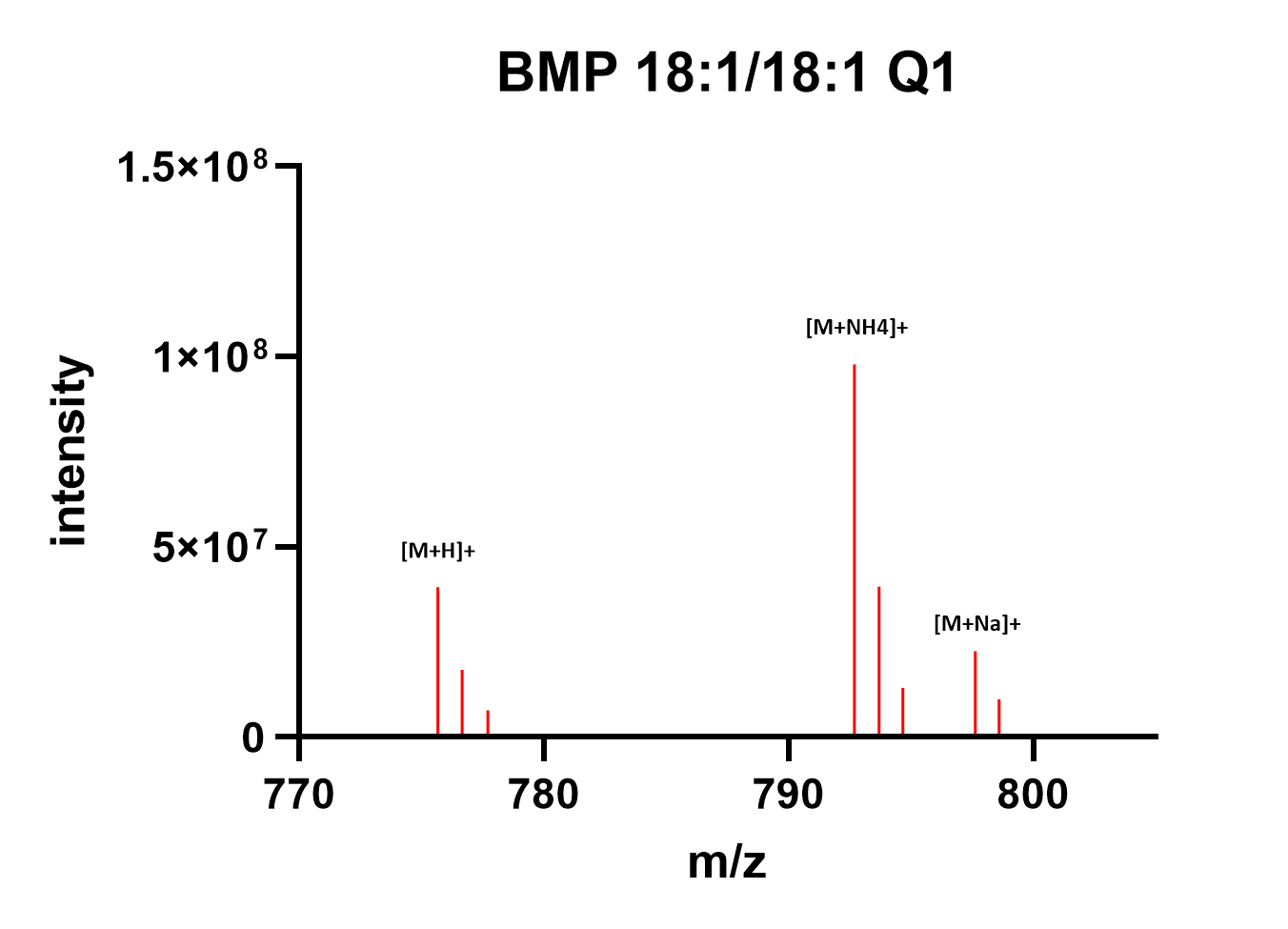


Figure S3: Various adduct ions formed by BMP 18:1/18:1 ESI+ mode.


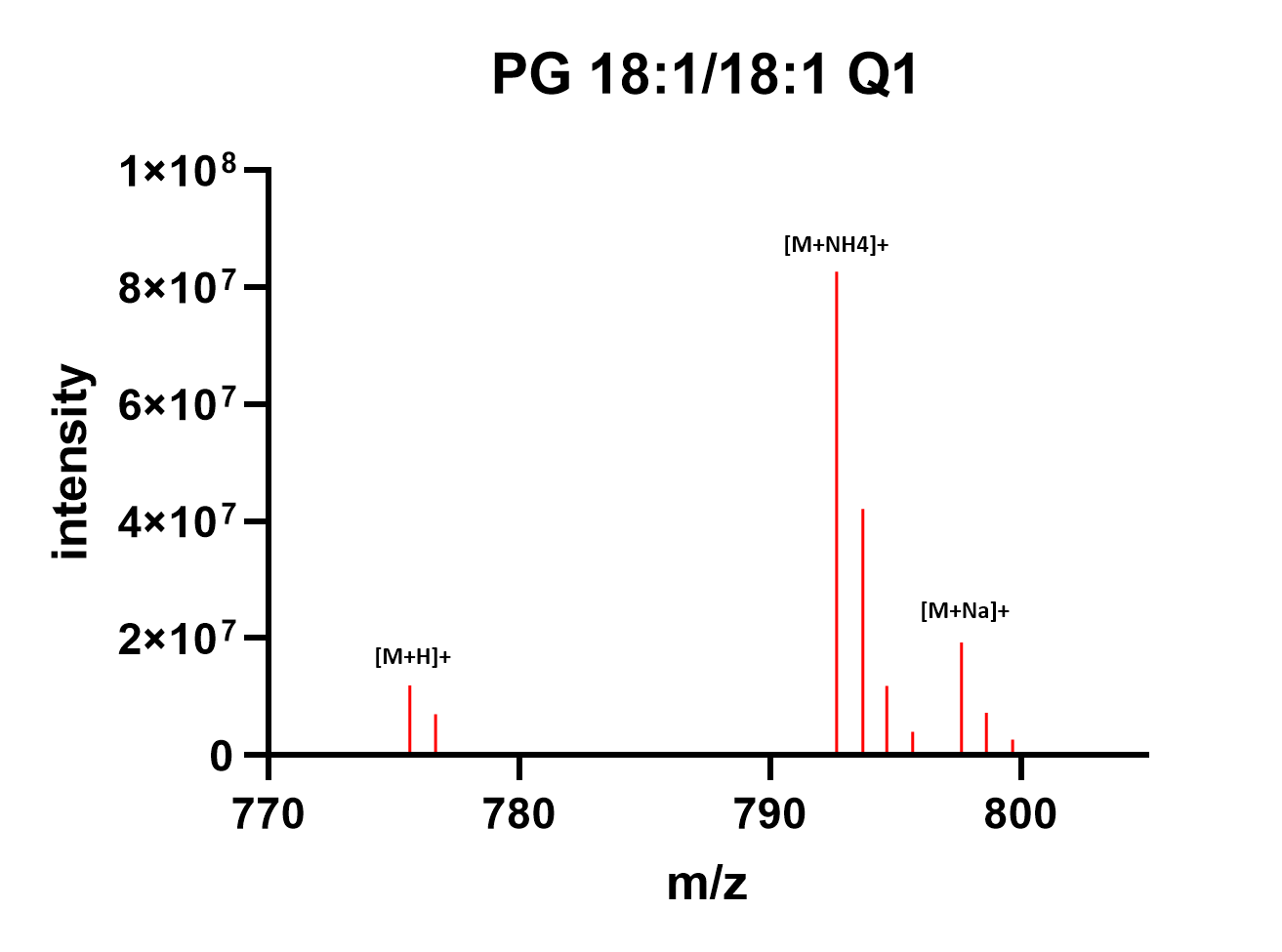


Figure S4: Various adduct ions formed by PG 18:1/18:1 in ESI+ mode.


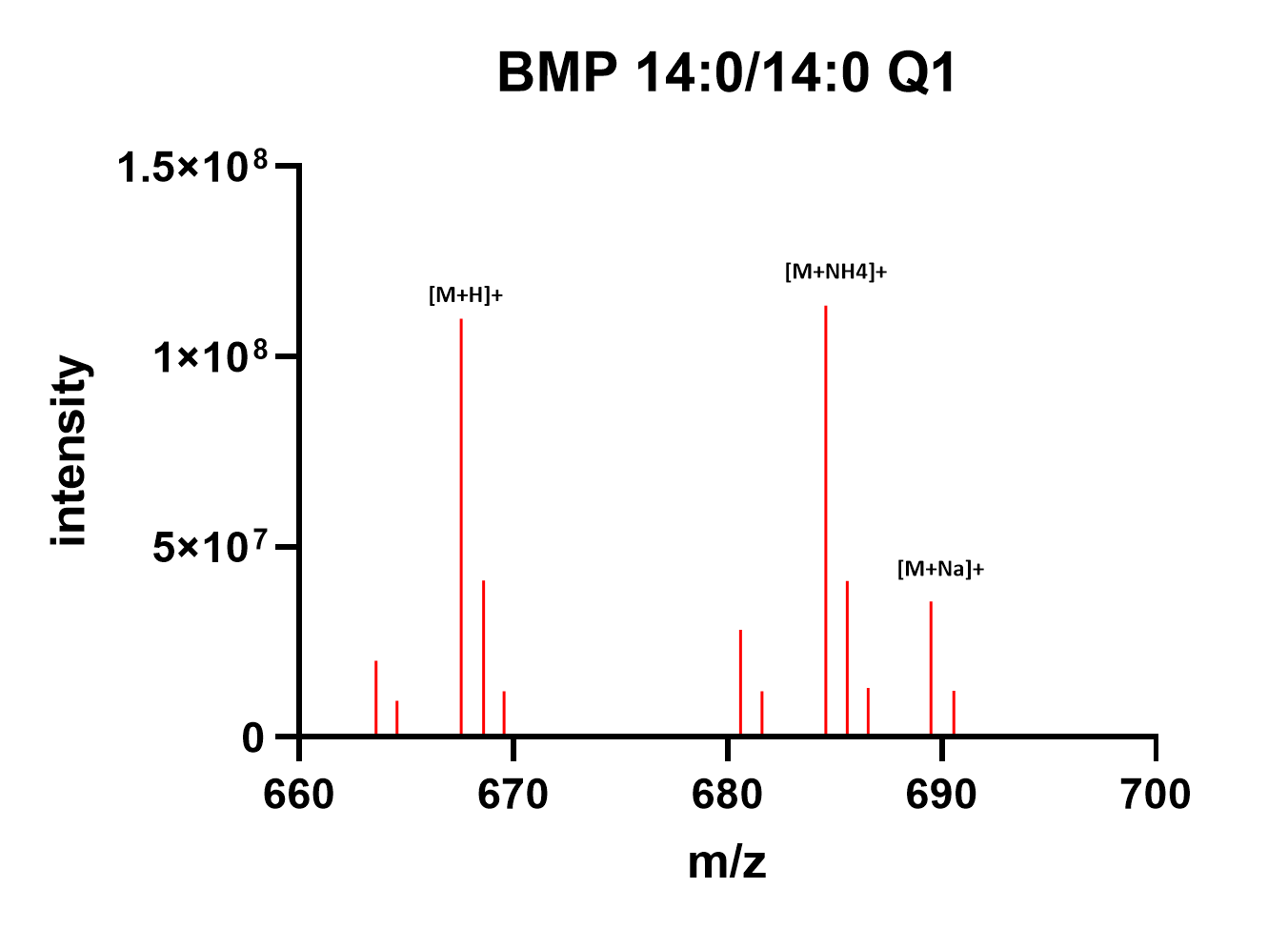


Figure S5: Various adduct ions formed from by BMP 14:0/14:0 in ESI+ mode.


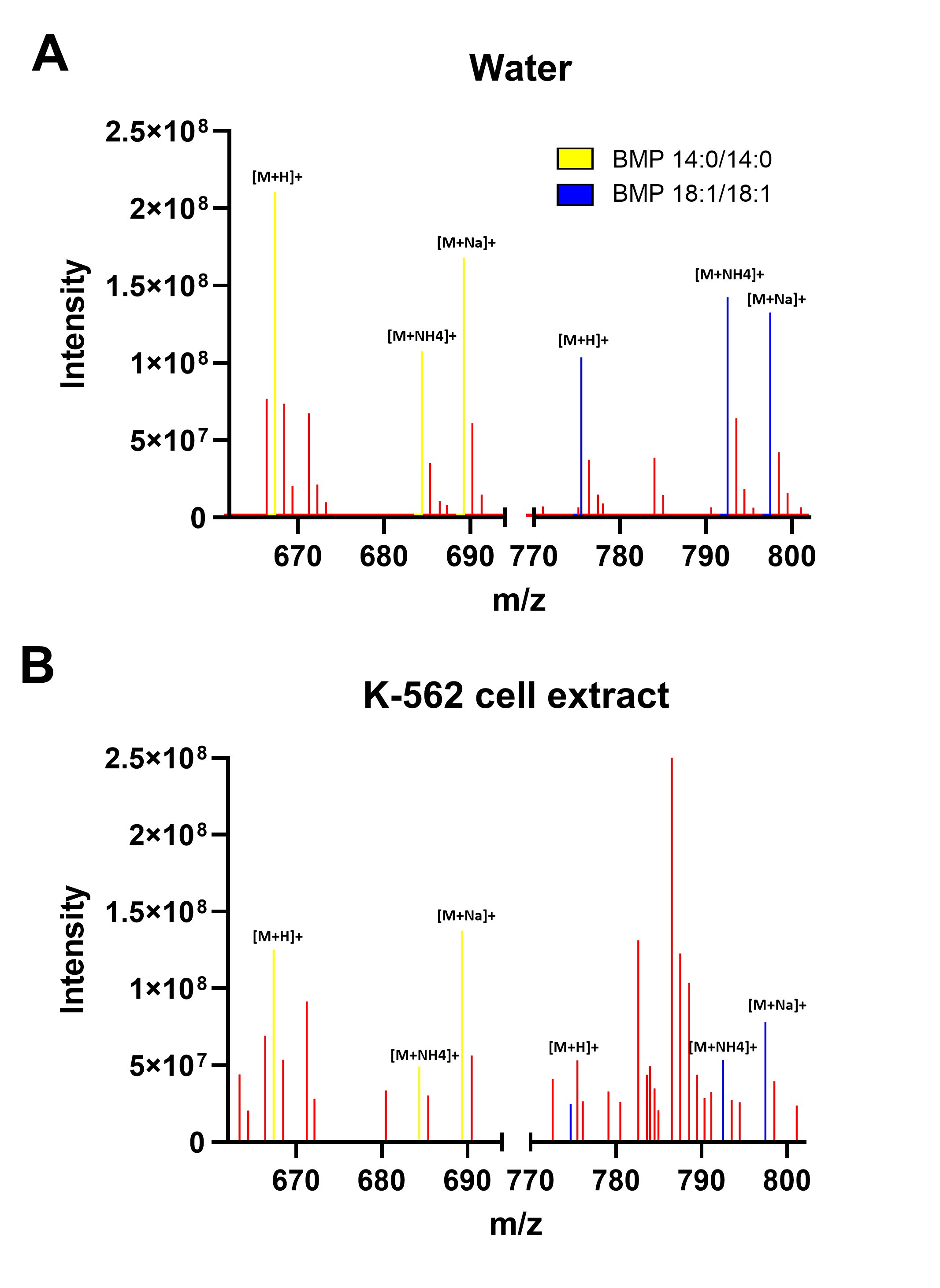


Figure S6: Ionization behavior of BMP 14:0/14:0 (16.7 µg/mL) and BMP 18:0/18:0 (16.7 µg/mL) when spiked in LC-MS grade water or K-562 cell extract.


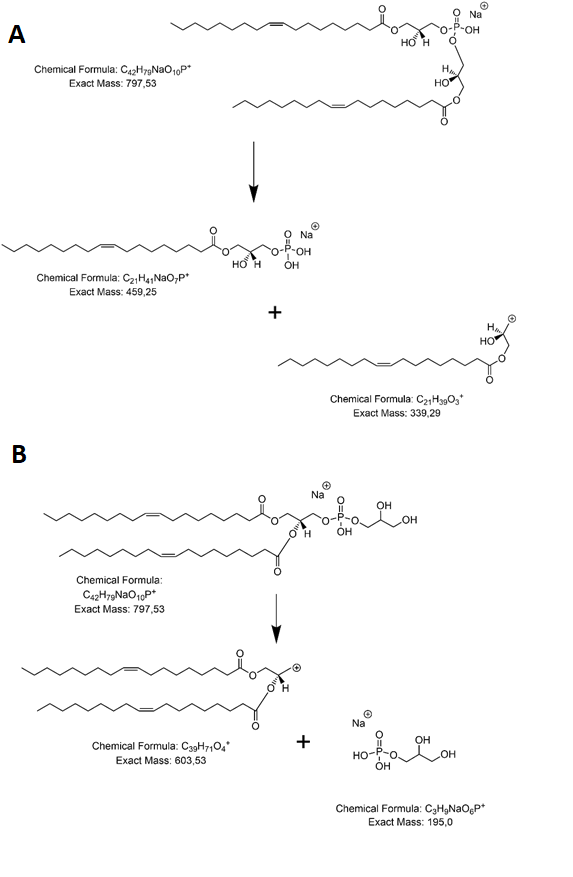


Figure S7: Schematic drawings of presumed sodiated fragmentation for BMP 18:1/18:1 (A) and PG 18:1/18:1 (B). Sodiated BMP 18:1/18:1 fragments into an unique mono-acyl fragment (*m/z* 459.25).


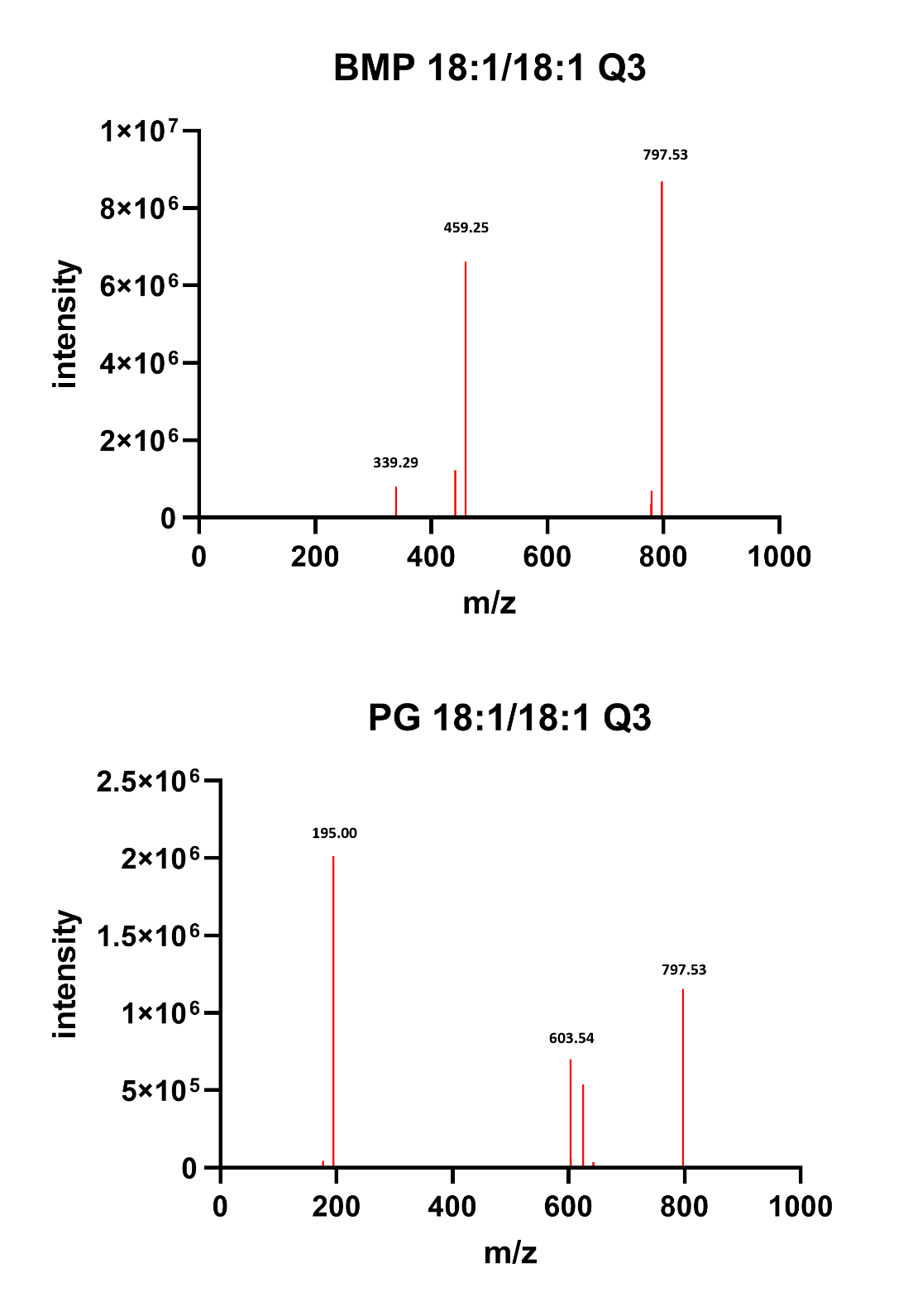


Figure S8: Fragments formed from sodiated BMP 18:1/18:1 ions (*m/z* = 797.53) and PG 18:1/18:1 ions (*m/z* = 797.53). Sodiated BMP forms a characteristic monoacyl fragment (*m/z* = 459.25)


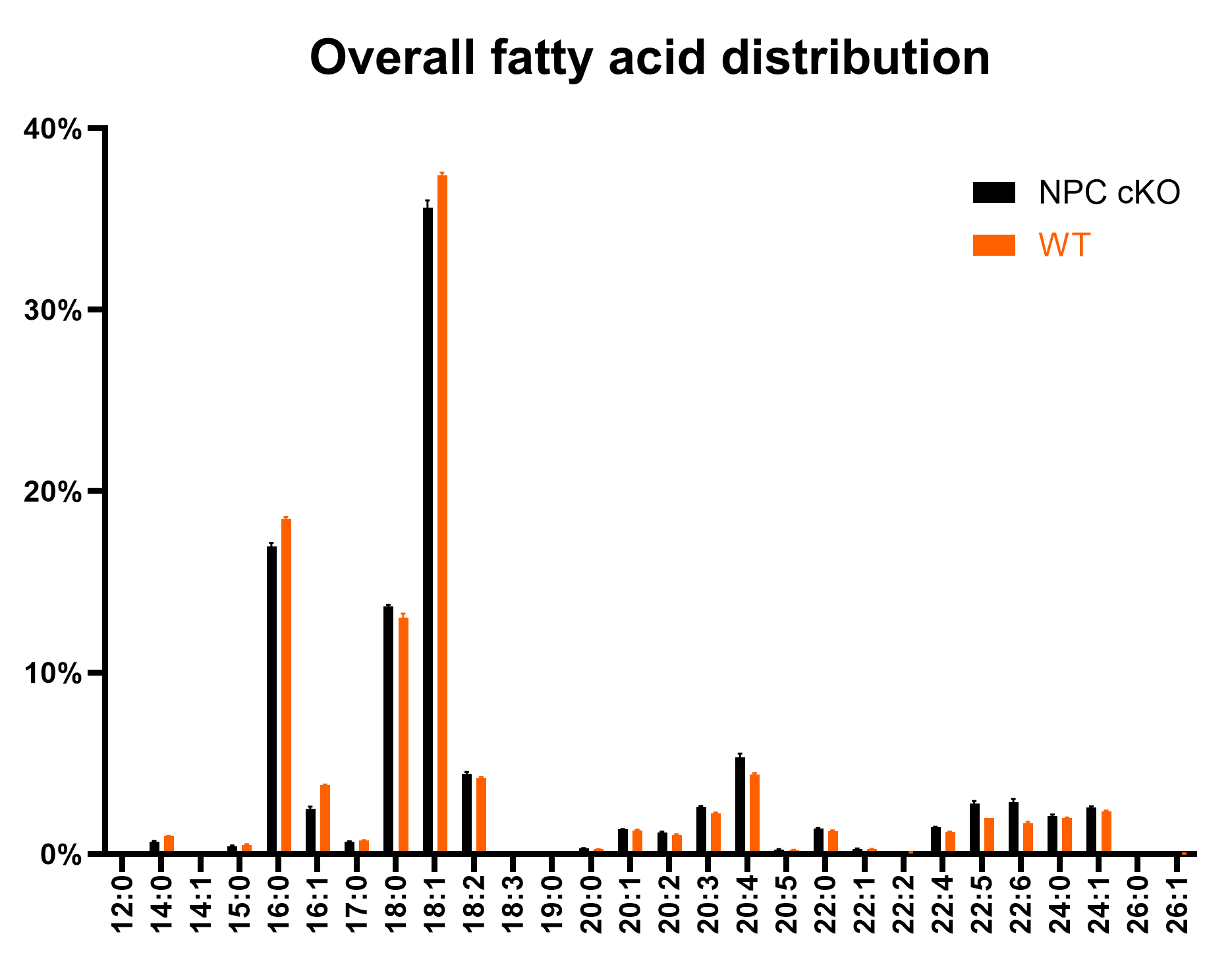


Figure S9: The distribution of FAs across all measured lipids NPC1 cKO and control.
